# Stent implantation in severe aortic coarctation in a pediatric patient with Turner syndrome: Case report and literature review

**DOI:** 10.3389/fped.2022.1041728

**Published:** 2023-01-26

**Authors:** Yuese Lin, Ling Zhu, Xuandi Li, Hongjun Ba, Xiufang He, Shujuan Li

**Affiliations:** ^1^ Department of Paediatric Cardiology, Heart Center, The First Affiliated Hospital, Sun Yat-sen University, Guangzhou, China; ^2^ Key Laboratory on Assisted Circulation, Ministry of Health, Guangzhou, China

**Keywords:** aortic coarctation, Turner syndrome, stent implantation, covered stent, complications

## Abstract

**Background:**

Turner syndrome is a rare systemic disease and a significant proportion of these patients experience aortic coarctation. Selection of optimal therapy for aortic coarctation in patients with Turner syndrome is difficult due to the pathologic change of the systemic vessel.

**Case presentation:**

We report one successful case of covered stent implantation for the treatment of severe native coarctation of the aorta in a 15-year-old patient with Turner syndrome weighing 36 kg. A covered stent was implanted in this patient. After the stent implantation, the peak systolic pressure gradient immediately decreased from 48 mmHg to 14 mmHg. The aortic diameter at the coarctation site increased from 3 mm to 10 mm after stenting. A femoral arterial complication occurred in this case, and we stabilized the situation finally.

**Results:**

During a follow-up of 3 years, no restenosis of aortic coarctation was observed and the patient no longer experienced hypertension. The dissection of the right femoral artery remained stable.

**Conclusion:**

A covered stent implantation for severe aortic coarctation in patients with Turner syndrome could be safe and effective. However, caution should be taken when using the technique to prevent complications.

## Background

Although stent implantation for the treatment of native coarctation of the aorta (CoA) has been widely reported in older children, adolescents, and adults, limited data exist on the results in patients with Turner syndrome (1). We report one case of a covered Cheatam-platinum stent implantation in severe aortic coarctation in a patient with Turner syndrome. Although a femoral arterial complication occurred in this case, we finally stabilized the situation. During a follow-up of 3 years, the patient remained asymptomatic and normotensive, and the dissection of the right femoral artery remained stable.

## Case presentation

**Figure 1:**
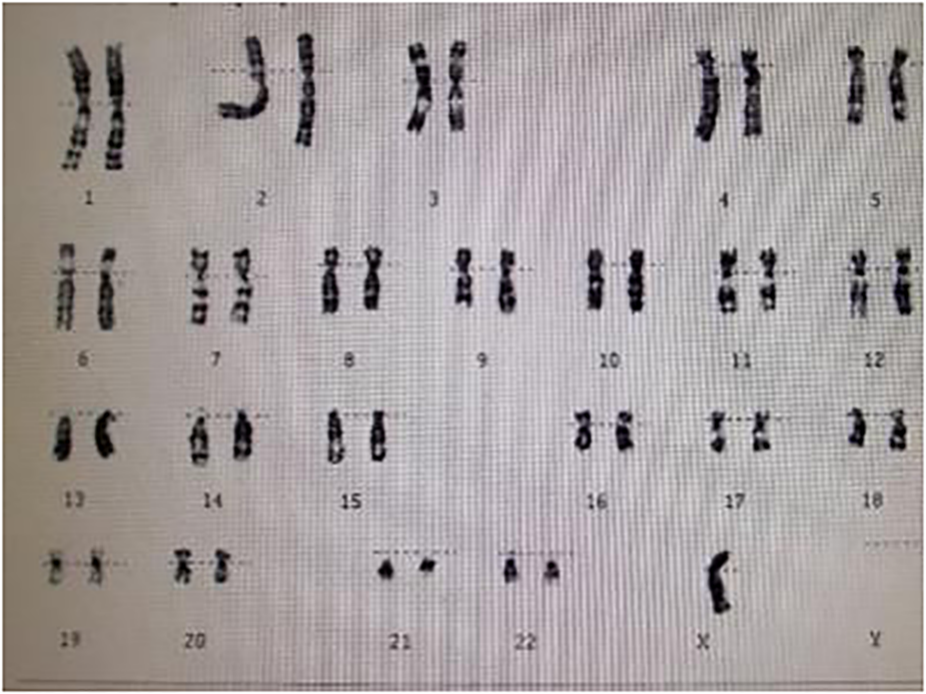
Karyotype analysis showing a pure 45, X karyotype.

A 15-year-old female patient was admitted to our hospital with a 12-year history of short stature and growth retardation. A physical examination on admission revealed that the weight and height of the patient was 36 kg and 1.37 m, respectively; both were less than three standard deviations of the same age. Special facial features of the patient were noted, including prominent posteriorly rotated auricles with looped helices, infraorbital skin creases, and a mildly foreshortened mandible. Other characteristic signs, including a webbed neck, low posterior hairline, and lack of breast development, were also found. The cuff blood pressure in both arms was 160/100 mmHg, and in both legs 110/80 mmHg. A cardiac examination demonstrated a grade 2/6 systolic ejection murmur in the second and third left intercostal spaces radiating to the suprasternal fossa and back. The chest x-ray was unremarkable. An ECG indicated cardiac hypertrophy and an echocardiography examination revealed a severe native aortic coarctation with a peak pressure gradient of 60 mmHg by Doppler image. The aortic valve was normal, and no signs of bicuspid aortic valve, aortic root dilatation, and dissection were found by echo. Renal ultrasonography was normal, but no obvious uterine and ovarian structures were found using gynecological ultrasonography. In view of these findings, a karyotype analysis was performed, and the result was a complete loss of the second X chromosome, revealing she had a pure 45, X karyotype (Figure 1). Based on these examination results, the patient was diagnosed with Turner syndrome with a severe native aortic coarctation.

**Figure 2:**
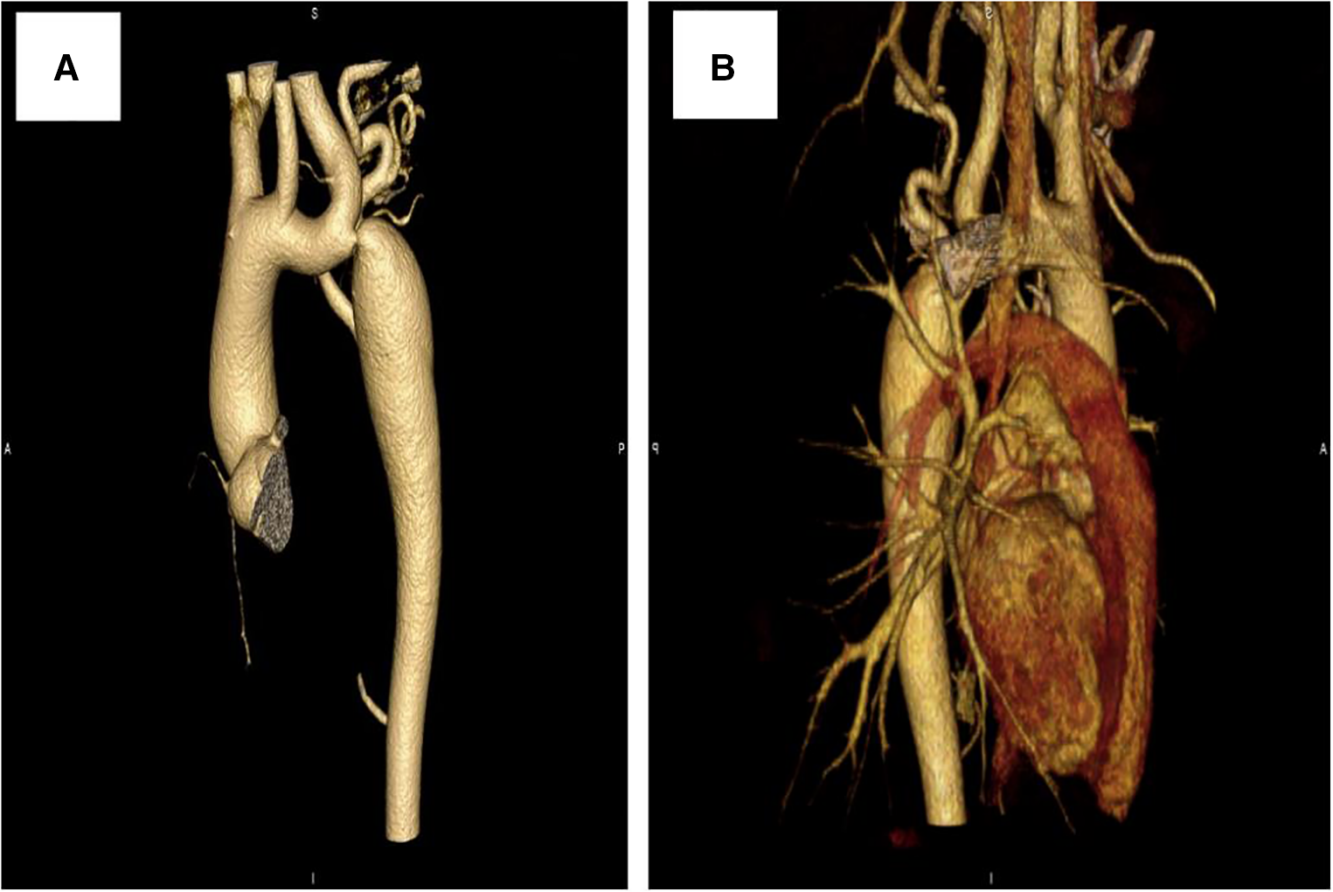
Computed tomography scan showing: (**A**) prior to stenting, (**B**) 3 years after stenting.

Further evaluations and examinations for cardiac malformation were performed. A CT scan showed a severe aortic coarctation just beyond the origin of the left subclavian artery (LSCA) (Figure 2A). At cardiac catheterization, under general anesthesia, the ascending aortic pressure was 138/85 mmHg and the descending aortic pressure was 90/72 mmHg. A descending aortogram confirmed a discrete lesion with a shelf-like in-folding and minimum diameter of 3 mm.

Due to the congenital dysplasia of the aortic wall and inherent vessel weakness in patients with Turner syndrome, and considering this was a case of severe aortic coarctation with the narrowest lesion being only 3 mm, we held a multidisciplinary team meeting for the case with surgeons and other colleagues to determine the appropriate choice of treatment. Finally, the interventional therapy of a covered stent implantation was considered to be suitable for this patient.

## Interventions and results

**Figure 3:**
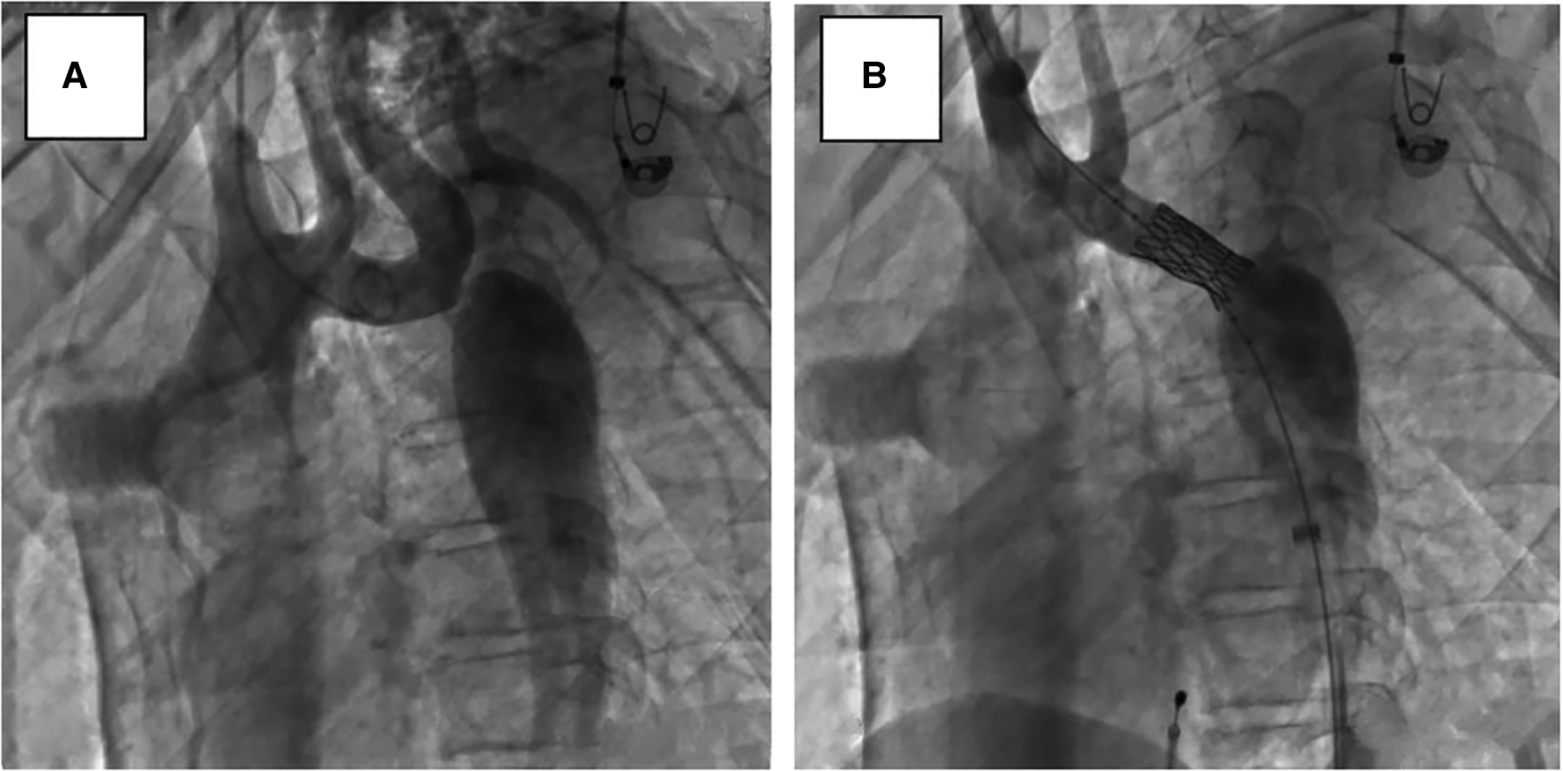
Lateral view, descending aortograms: (**A**) prior to stenting, (**B**) immediately after stenting.

**Figure 4:**
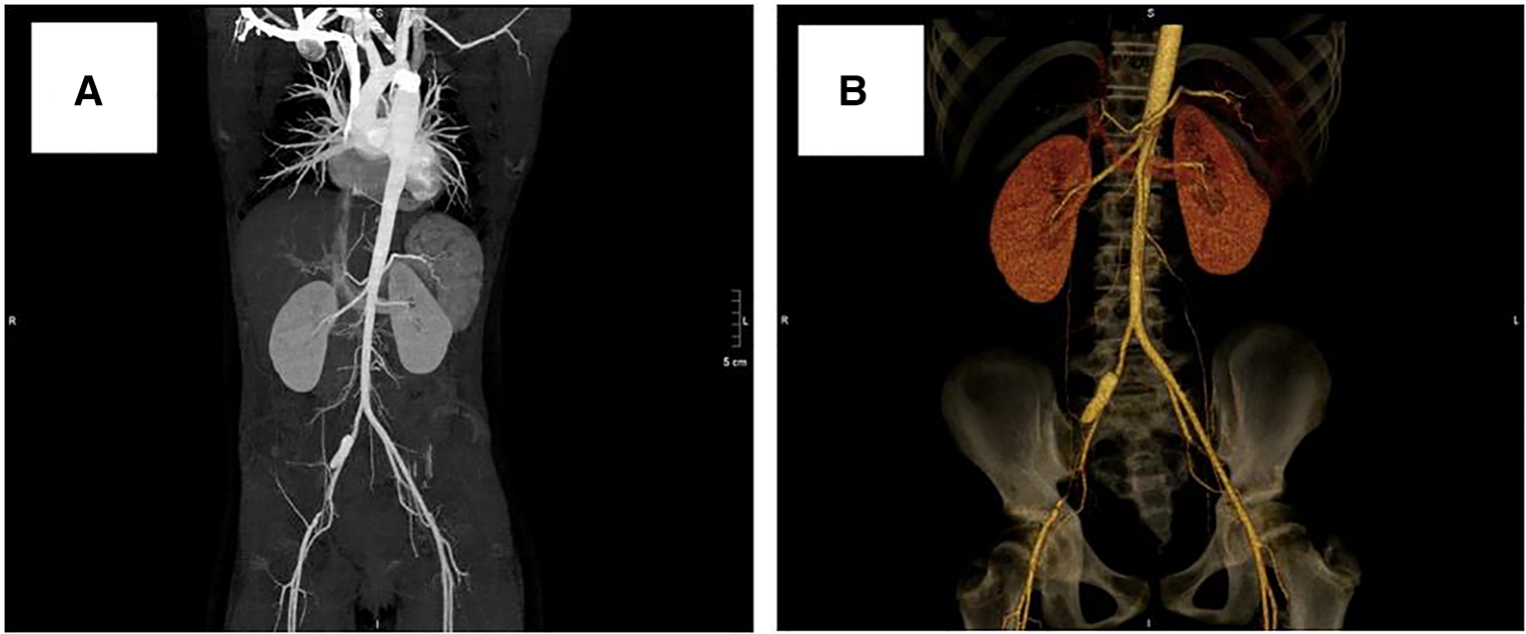
Computed tomography scan showing dissection of the right femoral artery: (**A**) 3 days after stenting, (**B**) 3 years after stenting.

After obtaining informed consent from the guardians, CoA stenting for the patient was performed very cautiously. Femoral arteriography showed that the diameters of both femoral arteries were similar, so we routinely picked the right femoral artery as the vessel access for stenting. The diameter of the aortic arch, thoracic aorta at the subclavian artery proximal to the coarctation site, and descending aorta at the level of the diaphragm were 11.0 mm, 11.8 mm, and 12.0 mm, respectively (Figure 3A). Therefore, we planned to expand the narrowest place to about 10 mm, which was not more than three times the diameter of the narrowest place and the diameter of the aortic arch, referring to the experiences and rules of stent implantation for severe CoA in the literature. A 22-mm-long covered Cheatam-platinum stent (NuMED, Hopkinton, NY, USA) mounted on a balloon in balloon catheter (NuMED), with a diameter of 12 mm and length of 25 mm, was implanted across the lesion through a 12-F delivery sheath. After stenting, the ascending aortic and descending aortic systolic pressure was 115 and 101 mmHg, respectively; the pressure gradient across the lesion decreased to 14 mmHg and a repeated descending aortogram showed that the diameter of the CoA increased to 10 mm, without angiographic evidence of an acute aneurysm formation (Figure 3B). However, 3 days after stenting, a CT scan showed a dissection of the right femoral artery (Figure 4A). Therapy with antihypertensive drugs and pain medication was initiated and the patient’s condition stabilized. The case was discussed with colleagues from the department of vascular surgery. It was judged whether a stent implantation in the femoral artery would be a suitable treatment if the patient’s condition deteriorated. Fortunately, during the next 2 weeks, the false lumen of the vessel did not widen further and perfusion of the right lower limb remained normal.

At a clinical follow-up visit 3 years after the stent implantation, the patient remained asymptomatic and normotensive, with a 10 mmHg gradient between upper and lower extremity blood pressures and a Doppler peak instantaneous gradient across the CoA of 15 mmHg by echocardiography. There was no evidence of aortic complications (Figure 2B) on CT scan and the dissection of the right femoral artery remained stable (Figure 4B). No signs of ischemia of the blood flow to the arterial side branches supplying the spinal cord and the left common carotid artery were observed during the follow-up.

## Discussion

Turner syndrome occurs in about 1 of 2,500–3,000 live female births, and approximately one-third of these patients experience a cardiovascular malformation. It is reported that 75% of these lesions have been a CoA or a bicuspid aortic valve with or without stenosis (2, 3). General treatment options for a native CoA have been surgery, balloon angioplasty, and stent implantation, depending on the age and weight of the patients, morphology and anatomy of the obstructive vascular lesion, and the local medical technology.

Surgical repair is the traditional management for CoA and the first choice of treatment in neonates and infants aged less than 3 months. Surgery also remains the only treatment option when transcatheter therapies fail. However, in 1984, it was already noted that surgery for aortic coarctation carries greater risks in patients with Turner syndrome (4). Zanjani et al. (5) summarized that the operative mortality and aortic wall injury (dissection, aneurysm formation) rates were 11% and 30%, respectively, after surgical repair of CoA in patients with Turner syndrome, which were obviously higher than those in genetically normal patients and also the highest among the above three treatment methods. Due to the inherent vessel wall weakness, namely cystic medial necrosis of the aortic wall, surgical repair may not be an ideal treatment option for a CoA in patients with Turner syndrome.

Balloon angioplasty has become an alternative treatment strategy for native and recurrent coarctation after surgery for many decades, with a good success rate and safety profile (6). Zanjani et al. (5) reported that balloon dilatation of CoA in patients with Turner syndrome carried the lowest mortality and risks of aortic wall injury among the three treatment options, which were 0% and 2%, respectively. Although balloon dilatation of CoA has excellent short-term results in patients with Turner syndrome (7), a high rate of restenosis after balloon angioplasty (8) and potential complications, including aortic dissection, aortic rupture, and aneurysm formation (9), have generated controversy regarding its use. Furthermore, it may result in a structurally weakened area instead of reconstructing normal vessel wall histology after balloon angioplasty (10), which could be the pathologic basis for the formulation of aneurysms and vessel recoil, which can lead to a recurrent lesion.

In past decades, endovascular stenting has been a safe and effective alternative treatment strategy to surgery or angioplasty in children and adults (11, 12). The mortality and aortic wall injury rates after CoA stenting in patients with Turner syndrome were reported as 6.6% and 20%, respectively (4), clearly lower than surgical repair. Stenting has theoretical advantages over balloon angioplasty: it not only prevents vessel recoil and maintains the effect of stent dilation, but also reduces the risk of aneurysm formation by preventing excessive dilation of the blood vessels (10). Still, aortic dissection after the stenting for native CoA has been noted (13). Fejzic et al. (9) reported a case of a bare metal stent implantation in a teenage patient with Turner syndrome; fetal aortic dissection occurred after a second procedure to redilate the stent. The patient's mother had previously died, also due to a dissection of the ascending aorta. A histological examination of the mother's aortic tissue showed fragmentation of elastic fibers and cystic medial necrosis. Further genetic study of the DNA examination demonstrated evidence of a mutation in the fibrillin-1 gene, similar to the changes found in Marfan syndrome. Shahri et al. (14) reported an aortic dissection occurred in nine patients who had received stent implantation therapy. Due to this inherent vessel wall weakness (15), use of a covered stent may be an advisable alternative treatment option in the setting of Turner syndrome. Covered stents can not only reduce the degree of traumatic injury to the aortic wall but also cover the injured wall in the stented area, which is especially appropriate and safe when there has already been aneurysm formation. In addition, as the pediatric patient grows older and develops physically, restenosis may occur at the primary CoA. In view of the above considerations, we selected a covered stent for this case. On the one hand, the covered stent could protect the blood vessel better and reduce the risk of complications, which is particularly meaningful to the vascular situation of patients with Turner syndrome. On the other hand, if restenosis occurs in the patient during follow-up, a second procedure to redilate the stent could be applied.

The most common significant complications of stent implantation were aneurysm formation, aortic dissection, cerebrovascular accident, and femoral access vessel injury. Additional concerns regarding the use of covered stents focus on the potential compromise of blood flow to the arterial side branches, especially those supplying the spinal cord, the head, and left upper limb, which may cause severe ischemia. In our case, the site of the CoA was just beyond the origin of the LSCA. As the stent was positioned to cover the CoA site, it would cross the LSCA but did not affect the blood flow to the spinal cord and the left common carotid artery. A CT angiography scan before the procedure confirmed the side branches supply the LSCA by the cerebral artery ring. With regard to femoral access vessel injury, however, it would be another serious complication after arterial catheter interventions. Shahri et al. (14) also reported that a dissection occurred in the external femoral artery among the nine patients after stenting. During the stent implantation of the native CoA, the diameter of the balloon is chosen to be equal to that of the normal aorta (usually in the transverse arch) and not greater than the diameter of the aorta at the diaphragm. The diameter of the delivery sheath should be 2 F larger than the balloon, and it is difficult to crimp over a balloon with a diameter less than 8–9 F (16). In our case, the size of the delivery sheath was12 F, which may be somewhat related to the occurrence of the femoral artery complication. Turner syndrome is a systemic disease, similar to Marfan syndrome, and the pathologic change of cystic medial necrosis would affect the blood vessels in the whole body. Femoral arteriography of both femoral arteries, to pick up the appropriate vessel access for stenting and to choose a smaller delivery sheath as far as possible during the procedure, is beneficial to reduce the risk of femoral artery complications.

Selecting the optimal therapy for CoA in patients with Turner syndrome is difficult due to the pathologic change of systemic vessel cystic medial necrosis. Although interventional treatment for CoA is a common treatment method and widely used in clinical practice nowadays, there are still limited data about the optimal choice for treatment, especially in patients with Turner syndrome. As we know, interventional therapy for severe aortic coarctation is challenging and somewhat difficult in clinical practice, especially when the pediatric patient has Turner syndrome. It not only increases the difficulty of the procedure itself, but also raises the risk of the operation, due to the inherent abnormality and weakness of the vascular wall. Taking into account the cases reported so far, stent implantation is not inferior to the other treatment methods with regard to mortality and vessel wall injury rates (5). Van den Hoven et al. (17) reported that adverse outcomes, such as aortic dissections, could occur in CoA in patients with Turner syndrome. However, there were only nine pediatric patients, and some adverse outcomes of cases were proved not to be related with the transcatheter procedure itself and no procedural complications were observed. A covered stent would be an interventional choice in the appropriate patient. Still, caution should be taken when using the technique and a larger population of pediatric patients is needed.

In conclusion, we presented a case of a successful covered stent implantation for the treatment of severe native CoA in a pediatric patient with Turner syndrome, with encouraging early and mid-term results, although not with a perfect outcome because of the femoral artery complication. A covered stent implantation could provide an alternative therapeutic option and could be safe and effective. However, vascular complications or adverse cardiac events may occur during the procedure and these should be handled cautiously. In addition, lifelong cardiovascular monitoring and follow-up for Turner syndrome is essential (1).

## Data Availability

The original contributions presented in the study are included in the article/Supplementary Material, further inquiries can be directed to the corresponding author/s.
